# Discovery and Flavor Characterization of High-Grade Markers in Baked Green Tea

**DOI:** 10.3390/molecules28062462

**Published:** 2023-03-08

**Authors:** Yushi Zou, Chen Tang, Xinyu Yang, Tianyang Guo, Huanlu Song

**Affiliations:** 1 School of Food and Health, Beijing Technology and Business University, Beijing 100048, China; 2 Department of Pharmacy, Beijing Shijitan Hospital, Capital Medical University, Beijing 100038, China

**Keywords:** baked green tea, odor, taste, metabolomics, GC×GC-O-MS, HPLC–MS

## Abstract

Green tea is a popular beverage around the world and possesses a unique flavor. The flavor qualities of green tea are closely related to its grade and this relationship has not yet been studied. Three baked green teas with similar flavor were studied, namely, Huangshan Maofeng, Taiping Houkui, and Shucheng Xiaolanhua. A total of 34 odor compounds were identified by solid phase microextraction (SPME) combined with two-dimensional comprehensive gas chromatography–olfactometry–mass spectrometry analysis (GC×GC-O-MS). The results of the clustering analysis showed that the content of D-limonene and linalool in the high-grade (Grade A) tea was much higher than the content in other grades, so they were identified as odor markers of Grade A baked green tea. The taste components of different grades of green tea infusion were analyzed by high-performance liquid chromatography–mass spectrometry (HPLC–MS) and HPLC. A combination of clustering analysis, principal component analysis (PCA), and orthogonal partial least squares discrimination analysis (OPLS-DA) indicated that galloylglucose, digalloylglucose, trigalloyglucose, strictinin, and gallic acid could be used as taste markers of Grade A baked green tea. Therefore, the results in this paper reveal the substances responsible for the odor and taste markers of high-grade baked green tea.

## 1. Introduction

Tea, which originated in China, has become one of the top three beverages in the world and is popular for its attractive flavor and health benefits [1]. Green tea is unfermented tea, and the original quality components in the tea plant’s fresh leaves are retained by the hyperthermal inactivation of certain enzymes [2]. According to different processing techniques, green teas are divided into baked green tea, half-pan-fried and half-baked green tea, pan-fried green tea, steamed green tea, etc. Baked green tea generally constitutes tea leaves that are treated via pan-fry killing and bake drying [3]. Common baked green teas include Huangshan Maofeng (H tea), Taiping Houkui (T tea), Shucheng Xiaolanhua (S tea), Jingting Baixue, and Tianshan Green Tea. With the qualities of a flowery or chestnut-like aroma and a fresh, brisk taste, baked green tea is popular with consumers. However, not all baked green tea can invoke pleasant feelings; only high-grade tea enables good sensory enjoyment.

According to the quality of the tea, tea can be divided into three grades [4]: Grades A, B, and C (i.e., “Jiaji”, “Yiji”, and “Bingji”, that is, high-grade, middle-grade, and low-grade, respectively) (shown in Table A1). Among them, Grade A green tea is described as having not only a mellow and thick as well as fresh and brisk taste but also a very fresh, chestnut-like/tender/orchid-like aroma. At present, the evaluation of tea quality is based on five factors: the taste of the infusion, the odor of the infusion, the color of the infusion, the shape of the dry tea leaves, and the characteristics of the tea leaves after infusion. Tea evaluators perform comprehensive grade assessments of tea using the “Methodology for sensory evaluation of tea (Chinese national standards, GB/T 23776-2018)” [4]. Among these factors, flavor, including odor (25% of the total score) and taste (30% of the total score), is the most critical quality criteria influencing a customer’s decision to purchase tea [5].

Traditionally, the discrimination of tea quality was mainly dependent on sensory evaluation and chemical detection. Among the diversiform techniques used to study the flavor of tea, GC-MS and LC-MS techniques were commonly used by researchers because they can individually identify the chemical compositions corresponding to the odor and taste in tea [6,7]. Modern instruments can be used to effectively detect the chemical composition of tea, and the flavor quality of tea is closely related to its chemical composition [8]. So far, about 260 volatile organic compounds (VOCs) have been isolated from green tea [9], and the VOCs with relatively high concentrations contribute greatly to the odor profile of tea. Among the VOCs in green tea detected by GC-MS, the top ten relative concentrations detected are as follows: geraniol; nerolidol; linalool; cis-3-hexenyl hexanoate; nonanal; 3,7-Dimethyl-1,5,7-Octatrien-3-ol; (E)-2-hexenal; phytol; heptanal; and (Z)-jasmone [9,10].

The tastes of green tea mainly include bitterness, astringency, umami, and a sweet after taste [11,12]. These tastes are provided by the water-soluble compounds in tea, including phenolic compounds [13], alkaloids [14,15,16], amino acids [17], nucleotides and organic acids, etc. Most taste compounds have been detected by a liquid chromatographic method. A total of 24 phenolic compounds of tea were detected by high-performance liquid chromatography combined with the ultraviolet wavelength (UV) and mass spectrometry (HPLC-UV-MS) [18]. An effective liquid chromatographic method that involved precolumn derivatization with o-phthaladehyde was developed for the determination of free amino acids in tea [19].

Teas of different quality grades are very similar in terms of their chemical components. However, they differ in terms of the concentrations of their chemical components [20,21]. Metabolomics is an effective tool used to determine the compounds associated with tea grades. For example, the volatile composition of Yichang big-leaf green tea (YBGT) was detected by headspace gas chromatography coupled with mass spectrometry (HS-GC-MS), and based on untargeted metabolomics, 16 volatile compositions were selected as characteristic markers for distinguishing low-grade from high-grade YBGT [6]. In addition, the metabolites of four different grades of Bai Mudan white tea were detected by ultra-performance liquid chromatography–quadrupole–time-of-flight mass spectrometry (UPLC-Q-TOF), and a model was constructed for the grade evaluation of white tea according to an untargeted metabolomics strategy [22]. Although there are some studies that have investigated the characteristic markers or key compounds in terms of odor or taste with which to distinguish low-grade from high-grade tea, there is still a dearth of comprehensive investigation of both the taste and odor compounds in tea, especially in a more general tea such as baked green tea, which is processed using very similar technology.

In this paper, metabolomics was applied to provide a scientific reference for the relationship between tea grading and flavor quality. Initially, baked green teas with similar flavors and quality grades (H tea, T tea, and S tea) were selected. The taste compounds and odor compounds in the infusions of the different grades of tea were determined by HPLC, HPLC–MS, and GC×GC-O-MS. The relationships between the flavor compounds and tea grades were studied by heat-map-clustering analysis. The grade markers in Grade A green tea were determined by PCA and OPLS-DA to provide a theoretical basis for the grading of baked green tea.

## 2. Results and Discussion

### 2.1. Analysis of Green-Tea-Screening Results

**Figure 1:**
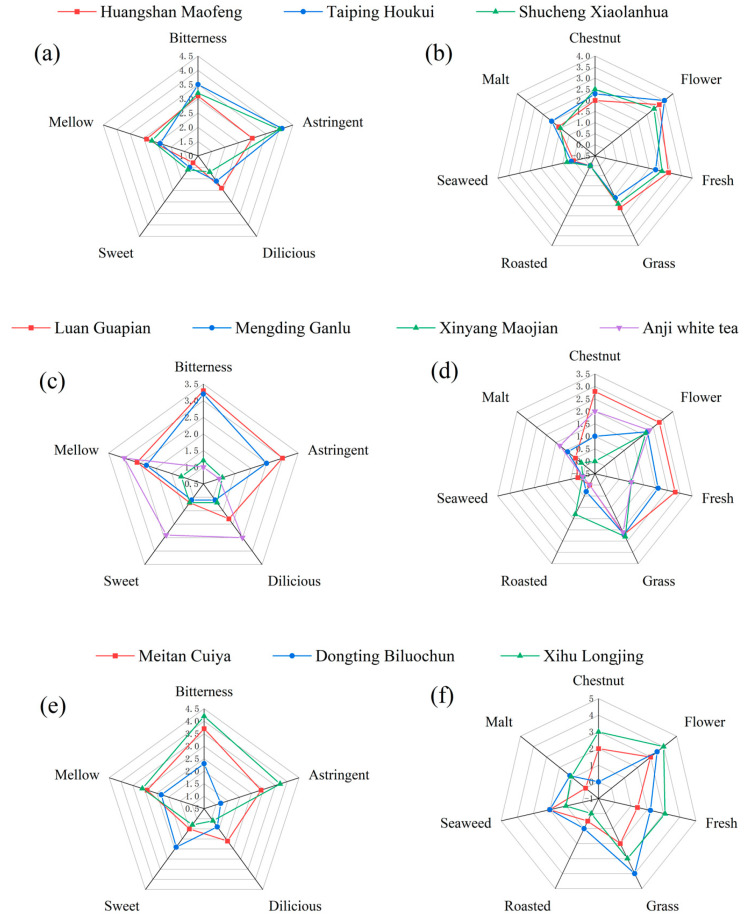
Radar diagrams of different kinds of green teas analyzed by sensory evaluation. (**a**) Taste evaluation of baked green teas. (**b**) Odor evaluation of baked green teas. (**c**) Taste evaluation of half-pan-fried and half-baked green teas. (**d**) Odor evaluation of half-pan-fried and half-baked green teas. (**e**) Taste evaluation of pan-fried green teas. (**f**) Odor evaluation of pan-fried green teas.

This study analyzed ten representative high-quality green teas, including baked green teas (Huangshan Maofeng, Taiping Houkui, and Shucheng Xiaolanhua), half-pan-fried and half-baked green teas (Luan Guapian, Mengding Ganlu, Xinyang Maojian, and Anji white tea), and pan-fried green teas (Xihu Longjing, Dongting Biluochun, and Meitan Cuiya). The tastes and odors of these teas were sensorily evaluated. A quantitative descriptive analysis of the green teas was used to determine differences in terms of the flavor quality of the different green teas. According to the taste radar diagram presented in Figure 1a, there was not much of a difference in the taste indicators between the three kinds of baked green tea, and their dominant taste was moderately mellow and brisk. In terms of odor, the results from Figure 1b show that all three kinds of baked green teas had characteristic odors, including chestnut-like, flowery, and fresh odors. Therefore, the types of baked green tea had similar flavor quality. The results of the sensory evaluation of the half-pan-fried and half-baked green tea are shown in Figure 1c,d; these results show that the four kinds of green tea differed significantly in terms of odor and taste. According to the results of the sensory evaluation of the pan-fried green tea in Figure 1e,f, the taste and odor of three kinds of pan-fried green tea also had relatively large differences. Finally, we selected these three kinds of baked green tea for inclusion in our following study.

### 2.2. Analysis of Baked Green Tea Grading Results

A total of 19 different commercially available baked green teas were selected and graded according to the sensory descriptions of Grade A, B, and C listed in the China National Standard (GB 23776-2018) [4]. The grading results are shown in Table 1, and the baked green teas were segregated according to their grades, as follows: four teas were categorized as Grade A, six as Grade B, and nine as Grade C. Among them, the Grade A baked green tea had the best flavor quality, exhibiting mellow and thick as well as fresh and brisk taste characteristics, and very fresh, chestnut-like, or tender/orchid-like aromas.

### 2.3. Odor Composition Analysis and Aroma Extract Dilution Analysis

SPME combined with two-dimensional comprehensive gas chromatography–olfactometry–mass spectrometry analysis (GC×GC-O-MS) was used to analyze the odor components in the different grades of baked green tea. A total of 34 odor compounds (specifically, 13 aldehydes, 6 esters, 4 ketones, 7 alcohols, 2 terpenoids, and 2 phenolic compounds) were identified in the 19 kinds of tea samples; 32 volatile compounds were identified in the different grades of H tea; 25 volatile compounds were identified in the different grades of T tea; and 28 volatile compounds were identified in the different grades of S tea. Through the calculation of the retention index (RI), the National Institute of Standards and Technology (NIST) database search, and an aroma extract dilution analysis (AEDA) [23], 22 odor active compounds were identified, including 2-methyl butyraldehyde, valeraldehyde, hexanal, (E)-2-pentenal, heptanal, (E)-2-octenal, furfural, (E,E)-2,4-heptadienal, benzaldehyde, β-cyclocitral, butyl hexanoate, cis-3-hexenyl hexanoate, ethyl acetate, (E)-methyl geranate, 1-penten-3-one, linalool, 3-octanol, hotrienol, α-terpineol, phenylethyl alcohol, β-myrcene, and D-limonene.

The odor compounds were extracted by SPME, and the contribution of each odor compound to each type of baked green tea was obtained by an odor dilution analysis of the odor active compounds in the three kinds of baked green tea samples via the AEDA method. The AEDA analysis revealed that the compounds contributing to the odor components of the three baked green teas were similar in composition, as shown in Table A2, thus rendering the odor profiles of the three green teas similar. A higher FD factor indicates a greater contribution to the overall odor [24,25]. It can be seen that linalool and β-myrcene, both with FD factors ≥27, are the compounds with the greatest odor contribution and have been shown to be the high-boiling-point compounds responsible for the formation of chestnut-like and flowery odors in the baking process [26,27].

### 2.4. Odor Composition Correlations Analysis and Grade A Odor Markers’ Identification

**Figure 2:**
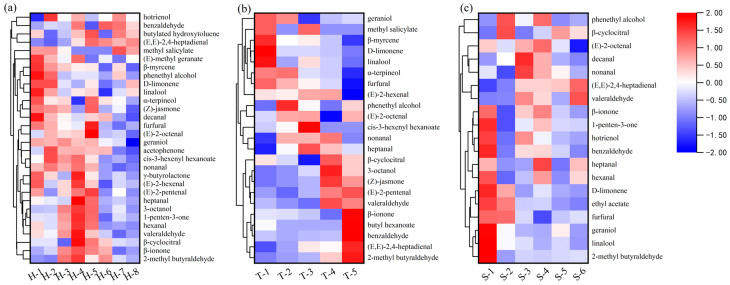
Heat-map-clustering analysis of odor compounds according to their concentrations in the baked green teas. Each square represents an odor compound. If a compound is present at a higher concentration, the color of the square becomes redder; otherwise, the color is bluer. (**a**) Huangshan Maofeng; (**b**) Taiping Houkui; (**c**) Shucheng Xiaolanhua.

The visualization results of the odor compounds in the different grades of baked green tea were obtained via cluster analysis of the relative quantification results of the odor compounds in the tea infusion of the three different kinds of H tea, T tea, and S tea (details shown in Figure 2). The odor compounds of the tea samples with different grades were very similar, but their odor concentrations were different. By comparing the color intensity variation of the heatmap in Figure 2, the Grade A tea (i.e., H-1, H-2, T-1, and S-1 tea) can be clearly distinguished from the other grades of tea. Through cluster analysis, it was determined that the Grade A tea had some similarities: D-limonene, linalool, and β-myrcene exhibited a pleasant odor and were found at nearly higher concentrations in the Grade A teas [28]; (E,E)-2,4-Heptadienal was found in lower concentrations in the Grade A tea, and it exuded a rather fatty odor [26,29]. The three kinds of teas also have their own characteristics of correlation in terms of odor. In the Huangshan Maofeng at Grade A and Grade B teas, (E)-2-pentenal and cis-3-hexenyl hexanoate were detected and characterized as pleasant odor compounds, while the difference between the Grade A tea and Grade B tea is that the Grade B tea also contained some unpleasant ingredients at higher concentrations, such as 2-methyl butyraldehyde, 1-penten-3-one, and 3-octanol. The presence of these compounds renders the odor of the Grade B tea infusion impure. Butyl hexanoate, which is unique in the type T tea, was higher in the Grade C tea and showed a negative correlation with grade. In Shucheng Xiaolanhua, ethyl acetate with a fruity odor was present at a higher concentration in the Grade A tea, while it was not identified in Huangshan Maofeng or Taiping Houkui.

**Figure 3:**
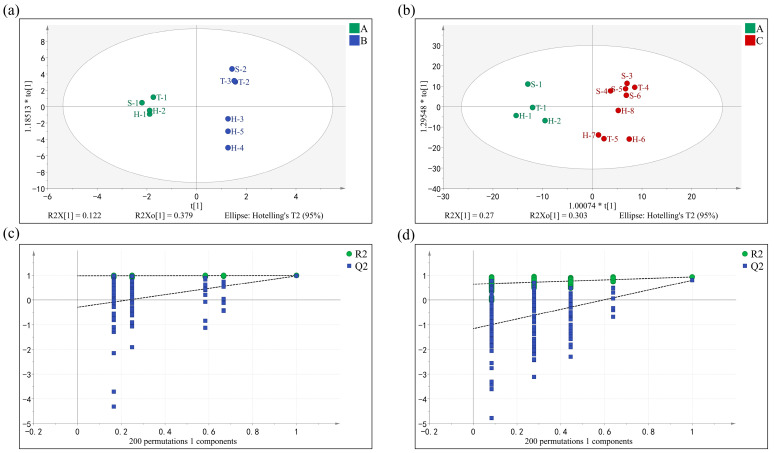
The OPLS-DA score plot of Grade A, B, and C baked green tea with odor compounds. (**a**) OPLS-DA score plots between Grade A and B tea: green color and blue color represent Grade A tea and Grade B tea, respectively. (**b**) OPLS-DA score plots between Grade A and C tea: green color and red color represent Grade A tea and Grade C tea, respectively. (**c**) Permutation test of OPLS-DA model between Grade A and B tea. (**d**) Permutation test of OPLS-DA model between Grade A and C tea.

OPLS-DA can eliminate data irrelevant to a category’s information through orthogonalization and screen out the characteristic variables of samples [30,31]. Figure 3a,b shows that the Grade A tea and other tea grades were clearly separated. R^2^ and Q^2^ values were used as indicators of model fitting and predictability, respectively [32]. The parameters of the OPLS-DA model were as follows: R^2^X = 0.994, R^2^Y = 0.926, and Q^2^ = 0.654 in the Grade A and B green tea samples; R^2^X = 0.815, R^2^Y = 0.949, and Q^2^ = 0.892 in the Grade A and C green tea samples; and R^2^ and Q^2^ over 0.5 indicate acceptable model fit results. After 200 permutation tests (results shown in Figure 3c,d), the intersection of the Q^2^ regression line with the vertical axis was less than zero, indicating that there was no overfitting of the model and, consequently, that the model was successfully validated. The results were considered to be applicable to the grade identification analysis of baked green tea odor compounds.

In conclusion, D-limonene and linalool were both important odor compounds in the three different kinds of baked green tea, and their concentrations in the grade A tea were much higher than those in the teas of grades B and C. In the sniffing experiment, D-limonene and linalool showed citrus-like and flowery aromas, respectively, which were both considered pleasant aromas. These two odor compounds were identified as odor markers of Grade A baked green tea.

### 2.5. Taste Composition Analysis and Correlation Analysis via Clustering Analysis

The composition and content of various non-volatile components in a tea infusion determine its unique taste quality; such components include phenolic compounds, alkaloids, amino acids, and so on. A total of 44 phenolic compounds were identified in the baked green tea via HPLC–MS. Phenolic substances are the main contributory components with respect to the astringency and bitterness of a tea infusion [15], and their content is a key quality indicator of tea. Amino acids are the main umami compounds of tea infusion; 14 amino acids in baked green tea were quantified by HPLC.

**Figure 4:**
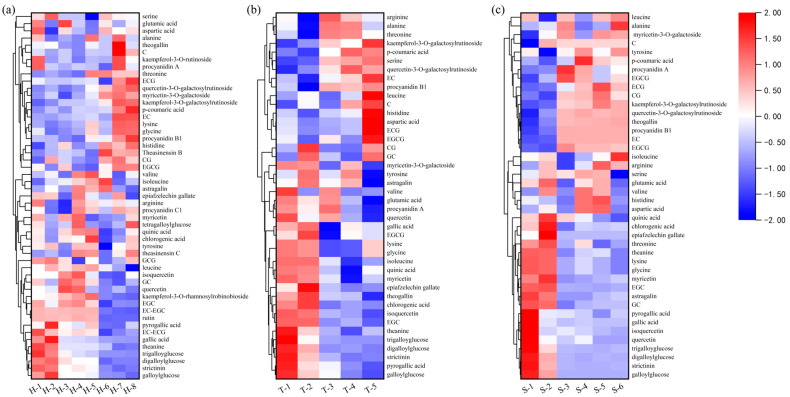
Heat-map-clustering analysis of taste compounds according to their content in the baked green tea. Every square represents a taste compound. If a compound has a higher concentration, the color of the square is redder; otherwise, the color is closer to blue. (**a**) Huangshan Maofeng; (**b**) Taiping Houkui; (**c**) Shucheng Xiaolanhua.

Based on the visualization results of the taste compounds of the different grades of H tea, T tea, and S tea shown in Figure 4, in the Grade A baked green tea, the concentrations of galloylglucose, digalloylglucose, trigalloyglucose, strictinin, and theanine were significantly higher than those in the grade B and C teas. EC, kaempferol-3-O-galactosylrutinoside, quercetin-3-O-galactosylrutinoside, and other taste compounds were found at lower concentrations in the grade A tea.

### 2.6. Identification of Grade A Taste Markers by PCA and OPLS-DA

**Figure 5:**
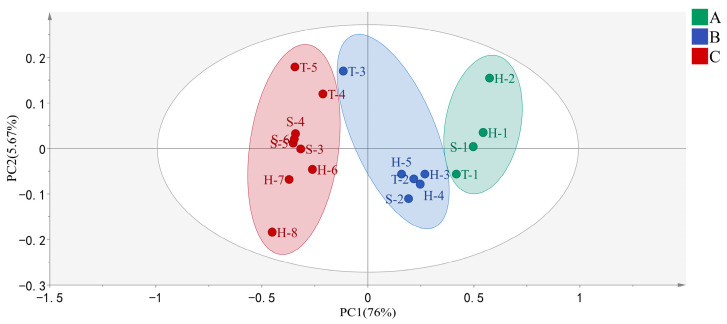
Forty-variable-based PCA of Grade A, B, and C baked green tea. The tea samples are located at the distinct positions in two-dimensional space described by two vectors of PC1 = 76% and PC2 = 5.67%. Green color represents Grade A tea, blue color represents Grade B tea, and red color represents Grade C tea.

PCA is a multivariate, unsupervised statistical analysis method used in metabolomics studies that can achieve dimensionality reduction and visually reflect some characteristics of samples [33]. As shown in Figure 5, the first two principal components (PC1 and PC2) accounted for 81.67% of the total variance, which indicated they were sufficient to explain most of the information of the sample. The separation of the different grades of tea was mainly based on PC1. A closer distribution of the tea samples shows more similar taste qualities. Therefore, these three different grades of baked green teas were distinguished via PCA. Upon comparison of the distances between samples, it is evident that the difference between grade A and grade C is large, while the difference between grade A and grade B is small.

**Figure 6:**
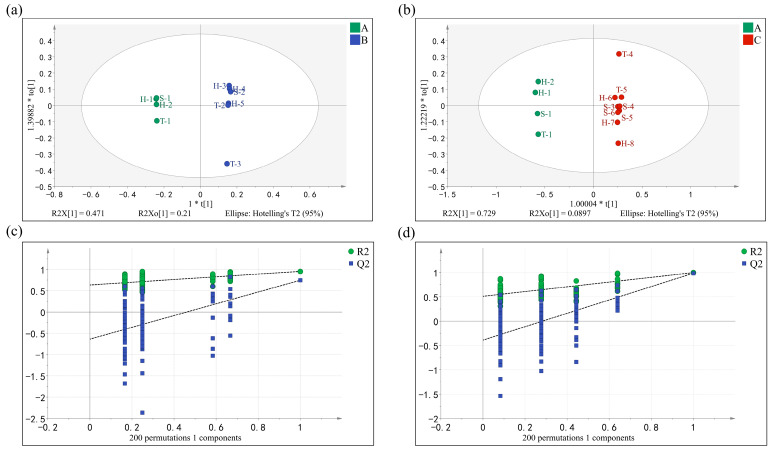
The OPLS-DA score plot of Grade A, B, and C baked green tea with taste compounds. (**a**) OPLS-DA score plots between Grade A and B tea: green color and blue color represent Grade A tea and Grade B tea, respectively. (**b**) OPLS-DA score plots between Grade A and C tea: green color and red color represent Grade A tea and Grade C tea, respectively. (**c**) Permutation test of OPLS-DA model between Grade A and B tea. (**d**) Permutation test of OPLS-DA model between Grade A and C tea.

The OPLS-DA model was constructed to further investigate the holistic characteristics and change correlations among the samples and identify the taste markers of Grade A green tea. Forty taste compounds in the tea infusion were used as dependent variables and different grades were used as independent variables to build an OPLS-DA model. The parameters of the OPLS-DA model were as follows: R^2^X = 0.945, R^2^Y = 0.999, and Q^2^ = 0.965 in Grade A and B green tea samples; R^2^X = 0.816, R^2^Y = 0.996, and Q^2^ = 0.981 in Grade A and C green tea samples; and R^2^ and Q^2^ over 0.5 indicate acceptable model fit results. The results of 200 permutation test was shown in Figure 6c,d, the model validation was effective. The results were considered to be applicable to the grade identification analysis of baked green tea taste compounds. The OPLS-DA score plot revealed that Grade A and other grades were clearly separated, as shown in Figure 6a,b.

**Figure 7:**
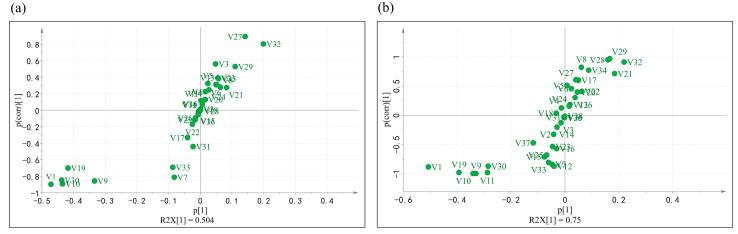
The S-plot of Grade A, B, and C baked green tea. (**a**) S-plot between Grade A and B tea. (**b**) S-plot between Grade A and C tea. V1-galloylglucose, V2-rutin, V3-theasinensin C, V4-tetragalloylglucose, V5-procyanidin C1, V6-EGCG, V7-GCG, V8-procyanidin B1, V9-strictinin, V10-trigalloyglucose, V11-EC-EGC, V12-quercetin, V13-GC, V14-EGC, V15-myricetin, V16-epiafzelechin gallate, V17-CG, V18-ECG, V19-digalloylglucose, V20-C, V21-EC, V22-theogallin, V23-chlorogenic acid, V24-astragalin, V25-isoquercetin, V26-myricetin-3-O-galactoside, V27-kaempferol-3-O-rhamnosyl-robinobioside, V28-theasinensin B, V29-quercetin-3-O-galactosylrutinoside,V30-gallic acid, V31-kaempferol-3-O-rutinoside, V32-kaempferol-3-O-galactosylrutinoside, V33-pyrogallic acid, V34-p-coumaric acid, V35-quinic acid, V36-caffeine, V37-EC-ECG, and V38-procyanidin A.

In Figure 7 shows the S-plot results of the differential metabolites in the different grades of the baked green tea samples. The dots at two ends of the “S” pattern indicate potential chemical markers with high confidence levels. In the S-plot of the A–B grades and A–C grades, it is obvious that there are five common taste compounds that were positively correlated with the Grade A tea at the bottom-left end of the “S” pattern, specifically, gallic acid, galloylglucose, digalloyglucose, trigalloylglucose, and strictinin.

Gallic acid, galloylglucose, digalloylglucose, trigalloyglucose, and strictinin are all phenolic compounds, and the concentrations of each of these compounds were significantly high in the Grade A tea. Therefore, combined with the results of the clustering analysis and OPLS-DA, they were identified as taste markers of Grade A Huangshan Maofeng, Taiping Houkui, and Shucheng Xiaolanhua tea. It is worth noting that galloylglucose, digalloylglucose, trigalloyglucose, and strictinin belong to the group of hydrolyzable tannins, and their content can be used as a potential indicator for high-grade green tea [34].

## 3. Materials and Methods

### 3.1. Samples

The different classes of H tea (8 classes), T tea (5 classes), and S tea (6 classes) originated from Huangshan in Anhui Province and were purchased from Yunchuan Tea Co., Ltd. (Hangzhou, China). The other seven kinds of green tea with high-quality, namely, Luan Guapian, Mengding Ganlu, Xinyang Maojian, Anji white tea, Xihu Longjing, Dongting Biluochun, and Meitan Cuiya, were purchased from their respective places of origin. All these green teas were sealed and stored in −18 °C freezer before sensory evaluation and odor/taste compound analysis.

### 3.2. Chemicals

Chromatographically pure acetonitrile and acetic acid was purchased from Thermo Fisher (Waltham, MA, USA). Catechins (C), epicatechin (EC), gallocatechin (GC), epigallocatechin (EGC), epigallocatechin gallate (ECG), gallocatechin gallate (GCG), epigallocatechin-3-O-gallate (EGCG), procyanidin B1, procyanidin C1, kaempferitrin, rutin, isoquercetin, myricetin-3-O-galactoside, myricetin-3-O-rutinoside, galloylglucose, gallic acid, cinnamic acid, chlorogenic acid, shikimic acid, p-coumaric acid, quinic acid, pyrogallic acid, theogallin, strictinin, theanine, theobromine, ethyl gallate, tannic acid, caffeine, L-sodium glutamate, and D-glucose were purchased from Yuanye (Shanghai, China). Quercetin, myricetin, kaempferol, and kaempferol-3-O-glucoside were purchased from Shanghai Zzstandard (Shanghai, China). Amino acid mixture standard reagent, borate buffer solution, o-phthalaldehyde (OPA), and 9-fluorene methyl chloroformate (FMOC) were purchased from Agilent Technology (Santa Clara, CA, USA). n-Hexane was purchased from Thermo Fisher (Waltham, MA, USA). 2-Methyl-3-heptanone and n-alkanes (C_7_–C_30_) were purchased from Sigma (St. Louis, MO, USA).

### 3.3. Sample Pretreatment

According to the China National Standard “Methodology for sensory evaluation of tea” (GB 23776-2018) [4], 2 g of tea was accurately weighed and placed in a 100 mL tasting cup, which was then filled with boiling water and covered for 4 min. Then, the tea infusion was filtered out at the same speed. The obtained tea infusion was to be employed for the sensory evaluation according to the odor and taste of the infusion.

For HPLC–MS and HPLC analysis, 1 mL of obtained tea infusion was filtered through a 0.45 mm membrane filter and loaded in 2 mL sample vial. For GC×GC-O-MS analysis, 15 mL of obtained tea infusion was loaded in a 40 mL headspace vial, and 1 μL of 2-methyl-3-heptanone (0.816 μg/μL) was added as the internal standard. Three replicates were performed for each sample for each detection method.

### 3.4. Sensory Evaluation

Bitter, astringent, umami, sweet, chestnut-like, flowery, fresh, grass, roasted, seaweed, and malt standards were configured into a series of standard liquids with concentration gradients as sensory strength standards. Bitterness, astringency, umami, and sweetness correspond to the following standards: caffeine, tannic acid, L-sodium glutamate, and D-glucose, respectively. The sensory evaluation team consisted of ten trained evaluators (five males and five females) and three tea evaluators who had national certificates for tea sensory evaluation. The ten evaluators were recruited from the Laboratory of Molecular Sensory Science at Beijing Technology and Business University and had at least one year of sensory training and a minimum of 100 h of general sensory testing including with respect to tea, coffee, milk powder, etc. The thirteen evaluators were familiar with green tea and were not allowed to use drinks, smoke, or drink/eat anything (except water) one hour before the sensory evaluation. Prior to the sensory evaluation, the participants were familiarized with the tea infusion to allow them to fully understand the China National Standard (GB 23776-2018) [4] and the taste and odor profiles were determined via description and discussion of the taste and odor of the tea infusion. The sensory evaluator compared the sensory results with the standards shown in Table A1 and recorded the score on the sensory evaluation form. These descriptors were scored from 0–5 in terms of intensity: 0, non-perceptible; 1, just perceptible/very weak; 2, weak intensity; 3, neutral intensity; 4, strong intensity; and 5, very strong intensity.

### 3.5. Odor Components Detection

#### 3.5.1. Solid-Phase Micro Extraction (SPME)

The tea infusion in vials were equilibrated in a constant-temperature water bath at 55 °C for 20 min. After 40 min extraction of odor compounds with DVB/CAR/PDMS fibers (50/30 μm, Supelco, Bellefonte, PA, USA) in a static state of the sample at 55 °C, the SPME fiber was inserted into the GC injector for desorption at 230 °C for 5 min.

#### 3.5.2. GC×GC-O-MS Detection of Odor Components

Qualitative analysis of odor compounds was performed according to the literature with modifications [35]. The extracted odor compounds were introduced in splitless mode into the Gas chromatographic instrument(Agilent 8890, Agilent Technologies, Santa Clara, CA, USA) and were simultaneously detected by Mass Spectrometer (Agilent 5975B, Agilent Technologies, Santa Clara, CA, USA) and a sniffing port (Sniffer 9100, Brechbuhler, Schlieren, Switzerland). A primary polar DB-wax capillary column (30 m × 0.25 mm, 0.25 μm film thickness; Agilent Technologies) was connected to a mid-polar DB-17 ms column (1.85 m × 0.18 mm, 0.18 μm film thickness; Agilent Technologies). The initial column temperature was set at 50 °C for a 5 min holding period; then, it was increased to 220 °C at a rate of 4 °C/min and maintained for a 4 min final holding period. Ultrahigh-purity helium was used as the carrier gas at a flow rate of 1 mL/min.

MS parameters were as follows: ion source temperature, 230 °C; transmission line temperature, 250 °C; quadrupole temperature, 150 °C; EI electron energy, 70 eV; mass range, 50–500; solvent delay, 4 min. The temperature of the sniffing port was 150 °C. Humid air was delivered into the pipeline of the sniffing port to maintain the sensitivity of the participants’ noses at all times. The samples were sniffed by three trained panelists and the time of the appearance of the smelled odor, its specific description, and the intensity of the odor were recorded.

The odor compounds were qualitatively analyzed by a combination of retention index (RI) from GC, ion peak from MS based on NIST14 library, and olfactory result (O) for comparative analysis. The RI values of the target compounds were calculated by referring to a previous work [35]. The relative concentrations of all odor compounds were obtained by semi-quantification method with 2-methyl-3-heptanone used as the internal standard.

### 3.6. Taste Components’ Detection

#### 3.6.1. HPLC–MS Detection of Phenolic Compounds

The qualitative and quantitative analysis of phenolic compounds was performed using high-performance liquid chromatography (Agilent 1200, Agilent Technologies, Palo Alto, CA, USA) coupled with QqQ mass spectrometer (Agilent G6410B, Agilent Technologies, Santa Clara, CA, USA). Preliminary detection was performed in full-scan mode and product ion mode of mass spectrometry for precursor ions and product ions; collision energy (CE) and fragment voltage were optimized using Optimizer software (Agilent Technology, (Santa Clara, CA, USA).

HPLC parameters were as follows: Mobile phase A was acetonitrile and mobile phase B was 0.1% formic acid water. A Zorbax Eclipse SB-C18 column (4.6 mm × 150 mm, 5 μm; Agilent Technologies, Santa Clara, CA, USA) was used. The injection volume was 10 μL, and the column temperature was set at 25 °C. The flow rate was set at 1 mL/min and the DAD detection wavelength was 280 nm. The following gradient was used (mobile phase A in B): 10% isocratic elution for 10 min from 10 to 40% (10–15 min), 40% isocratic elution (15–18 min) from 40 to 10% (18–20 min), and 10% reconditioning (20–25 min).

MS parameters were as follows: polarity, negative; capillary temperature, 300 °C; capillary voltage, 4000 V; drying gas temperature, 300 °C; drying gas flow rate, 6 L/min; nebulizer gas pressure, 55 psi.

The relative concentrations of all phenolic compounds were obtained using semi-quantitative method with ethyl gallate used as the internal standard. 

#### 3.6.2. HPLC Detection of Amino Acids

The amino acids in the samples were analyzed by HPLC (Agilent 1200, Agilent Technologies, Palo Alto, CA, USA) with a Zorbax Eclipse-AAA column (4.6 mm × 150 mm, 5 μm; Agilent Technologies, Santa Clara, CA, USA) through pre-column derivatization. Two standard solutions used were a mixture of 16 amino acids (i.e., Asp, Glu, Ser, His, Gly, Thr, Arg, Ala, Tyr, Cys, Val, Met, Phe, Ile, Leu, and Lys) and a L-theanine standard solution, and their concentrations were both 1 nmol/μL. HPLC parameters were as follows: mobile phase A, 0.04 mol/L NaH_2_PO_4_ H_2_O (pH 7.8); mobile phase B, methanol/acetonitrile/H_2_O (45/45/10, *v*/*v*/*v*); temperature, 40 °C; flow rate, 1.5 mL/min; and DAD detection wavelength, 338 nm. Automated derivatization procedure: 2.5 μL of borate buffer solution was mixed into 0.5 μL of standard solution or sample solution; then, 0.5 μL portions of OPA and FMOC were added, respectively, and they were all mixed uniformly 6 times; finally, 32 μL of ultrapure water was pipetted and 20 μL of above-mixed solution was injected. The following gradient was used (mobile phase A in B): 100% isocratic elution for 1.9 min, from 100 to 43% (1.9–18.1 min) and then from 43 to 0% (18.1–18.6 min); 0% isocratic elution (18.6–22.3 min) from 0 to 100% (22.3–23.2 min); and 100% reconditioning (23.2–26 min). The external standard method was adopted for quantitative analysis.

### 3.7. Data Analysis

The data from HPLC–MS were processed by MassHunter Qualitative and quantitative Analysis B.07.00 software (Agilent Technologies, Santa Clara, CA, USA); the data from GC×GC-O-MS were processed by Canvas software (Xuejing Electronic Technology, Shanghai, China); and the data from HPLC were processed by Chemstation software (Agilent Technologies, Santa Clara, CA, USA). Heat-map-clustering analysis of data was performed using Origin 2021b software (OriginLab, Northampton, MA, USA). PCA and OPLS-DA of data were performed using Simca-P 14.1 software (Umetrics, Malmö, Sweden).

## 4. Conclusions

Green tea is popular among consumers worldwide due to its great flavor, but the relationship between its grades and flavor compounds remains unclear. In this study, baked green teas with similar flavors (i.e., Huangshan Maofeng, Taiping Houkui, and Shucheng Xiaolanhua) were selected as research objects by sensory evaluation. The relationship between grades and flavor compounds was investigated, and the markers of Grade A baked green teas were identified. Regarding odor, the odor compounds in the infusion of different grades of baked green tea were analyzed by SPME-GC×GC-O-MS, and a total of 34 odor compounds and 22 odor active compounds were identified in 19 tea samples via sniffing. Through further analysis by AEDA dilution and data visualization, D-limonene and linalool were identified as Grade A baked green tea odor markers. With regard to taste, HPLC–MS was used to analyze the taste compounds in the infusion of different grades of baked green tea; consequently, a total of 44 phenolic compounds and 14 amino acids were identified in 19 tea samples. The PCA and OPLS-DA were used to statistically analyze the data. The results showed that the concentrations of gallic acid, galloylglucose, digalloylglucose trigalloyglucose, and strictinin were significantly higher in the Grade A tea than in the other grades. This result indicated that the teas were significantly different between groups and identified as the taste markers of Grade A baked green tea.

The study provides a scientific reference for baked green tea grading and odor and taste markers of Grade A baked green tea; these findings can be used as the basis for judging the quality of baked green tea. Our findings will need to be verified and supplemented in the future to ensure the richness and comprehensiveness of baked green tea samples.

## Figures and Tables

**Table 1:** Grades A, B, and C of baked green tea determined via sensory evaluation grading results.

Sample ^1^	Market Grade ^2^	Abbreviation	Score ^3^	Grade ^4^
H tea	Best Class	H-1	92 ± 0.3	A
	Super class Grade A	H-2	96 ± 0.5	A
	Super class Grade B	H-3	85 ± 0.2	B
	Super class Grade C	H-4	82 ± 0.4	B
	Super class	H-5	83 ± 0.3	B
	First class Grade A	H-6	75 ± 0.8	C
	First class Grade B	H-7	76 ± 0.9	C
	Second class	H-8	72 ± 0.4	C
T tea	Best Class	T-1	93 ± 0.1	A
	Super class Grade A	T-2	87 ± 0.5	B
	Super class Grade B	T-3	84 ± 1.1	B
	Super class	T-4	74 ± 0.3	C
	First class	T-5	73 ± 0.2	C
S tea	Super class Grade A	S-1	94 ± 0.2	A
	Super class Grade B	S-2	83 ± 0.7	B
	First class	S-3	74 ± 0.4	C
	First class Grade A	S-4	75 ± 0.6	C
	First class Grade B	S-5	72 ± 0.3	C
	First class Grade C	S-6	78 ± 0.2	C

^1^ H tea: Huangshan Maofeng; T tea: Taiping Houkui; S tea: Shucheng Xiaolanhua. ^2^ The quality of each tea worsens from top to bottom. For example, ‘Best Class’ is the best and ‘Second Class’ is the worst with respect to H tea. ^3^ A total of 13 evaluators have made evaluation from a 100-point scale (0~100 points), and each sample score was averaged. ^4^ According to the five factors of tea evaluation in China National Standard “Methodology for sensory evaluation of tea” (GB 23776-2018), different market grades of teas were divided into Grades A, B, and C (i.e., “Jiaji”, “Yiji”, and “Bingji”, which denote high-grade, middle-grade, and low-grade, respectively).

## Data Availability

Data are shown in the article.

## Appendix A

**Table A1:** Green tea quality characteristics describe score.

Factor	Grade	Quality Characteristics	Score
Taste	A	sweet and fresh or fresh and mellow, heavy and mellow, mellow and thick, fresh and brisk	90~99
	B	clean and brisk, strong and slightly mellow, slightly mellow and thick	80~89
	C	slightly mellow, strong and astringent, grassy and astringent	70~79
Odor	A	high fresh, chestnut-like/tender/orchid-like aroma	90~99
	B	fresh, tender or high-fired aroma	80~89
	C	less pure, dull odor, over-fired aroma	70~79

## Appendix B

**Table A2:** AEDA dilution analysis results of the highest class of H, T, and S tea by GC×GC-O-MS.

Odor-Active Compound	Identification Method	Odor	log_3_ FD		
			H-1 Tea	T-1 Tea	S-1 Tea
2-methyl butyraldehyde	MS/RI/O	cocoa	1	1	1
1-penten-3-one	MS/RI/O	peppery	1	-	1
hexanal	MS/RI/O	grass	3	-	3
3-octanol	MS/RI/O	mushroom	2	1	1
phenethyl alcohol	MS/RI/O	floral	2	2	2
linalool	MS/RI/O	floral, lavender	3	3	3
(E)-2-pentenal	MS/RI/O	fruity	1	-	-
(E)-2-octenal	MS/RI/O	nut	1	1	1
furfural	MS/RI/O	sweet, baked bread	1	1	1
benzaldehyde	MS/RI/O	bitter almond	1	1	2
β-cyclocitral	MS/RI/O	herbal	2	2	2
hotrienol	MS/RI/O	floral	1	-	2
D-limonene	MS/RI/O	citrus orange	1	2	2
β-myrcene	MS/RI/O	balsamic	3	3	-
butyl hexanoate	MS/RI/O	fruity	-	1	-
α-terpineol	MS/RI/O	pine	1	1	-
cis-3-hexenyl hexanoate	MS/RI/O	fruity	1	1	-
(E)-methyl geranate	MS/RI/O	refreshing	1	-	-
ethyl acetate	MS/RI/O	fruity	-	-	2
(E,E)-2,4-heptadienal	MS/RI/O	fatty	1	1	1
valeraldehyde	MS/RI/O	malt	1	1	1
heptanal	MS/RI/O	citrus	1	1	1
